# Supplementary material for: Local hippocampal fast gamma rhythms precede brain-wide hyperemic patterns during spontaneous rodent REM sleep
Source: Nat Commun. 2018 Dec 18;9:5364. doi: 10.1038/s41467-018-07752-3 (PMC6299136; doi:10.1038/s41467-018-07752-3)
Supplement: Supplementary file 2 — Supplementary Information [file 41467_2018_7752_MOESM2_ESM.pdf]

Local hippocampal fast gamma rhythms precede brain-wide hyperemic patterns during spontaneous rodent REM sleep

A. Bergel et al.

Supplementary Material

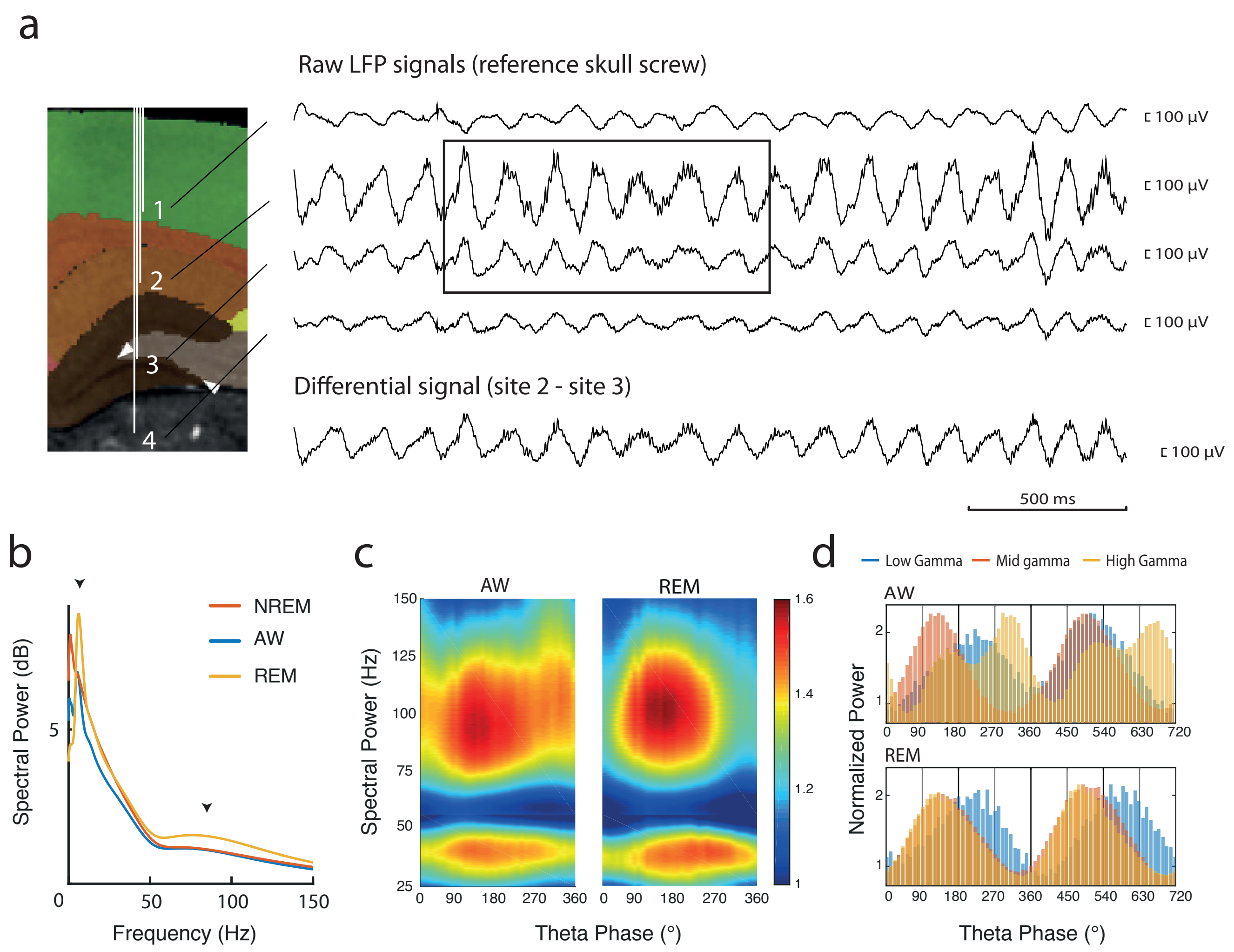

Supplementary Figure 1: Electrophysiological recordings in the dorsal hippocampus

(a) Electrodes bundles (white) overlaid on the Waxholm Atlas of the Rat Brain at Bregma = - 4.0 mm. Adapted from reference [1], Copyright (2015), with permission from Elsevier. Targeted structures are parietal association cortex (Green - 1), CA1 region (Light Brown - 2), dentate gyrus (Dark Brown - 3) and dorsal thalamus (Grey contrast - 4). Details of REM theta oscillations for raw LFP traces at each recording site. We computed differential signals between CA1 and dentate recording sites to obtain the best signal to noise ratio.

(b) Average power spectral density for the differential signal shown in (a). REMS is characterized by sustained power in the theta and mid gamma bands (arrows). AW is characterized by theta power while NREM shows stronger activity in the delta band (1-4 Hz) and flat distribution over other frequency bands.

(c) Wavelet based theta-phase spectrogram showing 3 different gamma sub-bands during AW and two different gamma sub-bands during REM sleep[2].

(d) Low-Gamma (20-50 Hz) Mid-Gamma (50-100 Hz) and High-Gamma (100-150) Hz power show theta phase modulation between AW and REM. Note the 90°-shift between slow gamma and mid-gamma power peaks. High-gamma during AW shows two distinct peaks around theta peak (180°) and theta trough (0°).

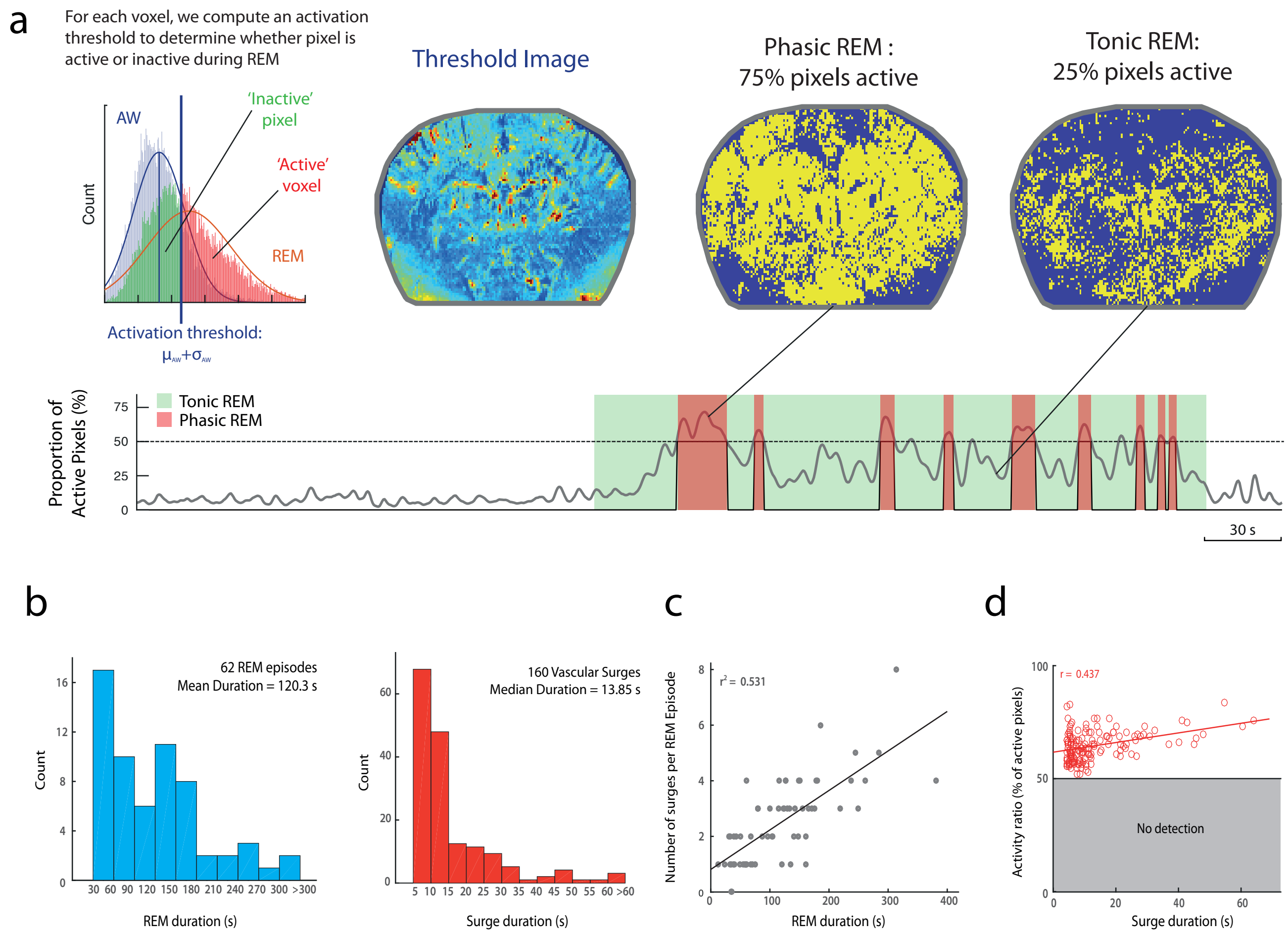

Supplementary Figure 2: Details of phasic/tonic REM sleep classification

(a) Phasic REM epochs (vascular surges) are detected by first extracting AW distributions for each pixel in an image and computing the mean μ and standard deviation σ. A threshold image is formed by computing $\mu+p\sigma$ for each pixel, where p is the first parameter (activation threshold). A pixel is thus considered active if the CBV level is above activation threshold. A second parameter m, the extent threshold, is set as the minimum proportion of active pixels over the total number of brain pixels, to discriminate between phasic and tonic regimes. We typically used p=1 and m=0.5. We show two typical images during phasic and tonic REM with active pixels in yellow and silent pixels in blue.
(b) REM episodes and surges distributions for all recordings (7 animals), for a total of 62 REM episodes and 160 vascular surges.
(c) Relationship between REM episode duration and the number of surges per episode, showing a clear positive correlation.
(d) Relationship between surge extent (the maximum proportion of active pixels during a surge) and surge duration. Weak correlation between these two variables, owing to the fact that short surges (<15 s) show variability in surge extent.

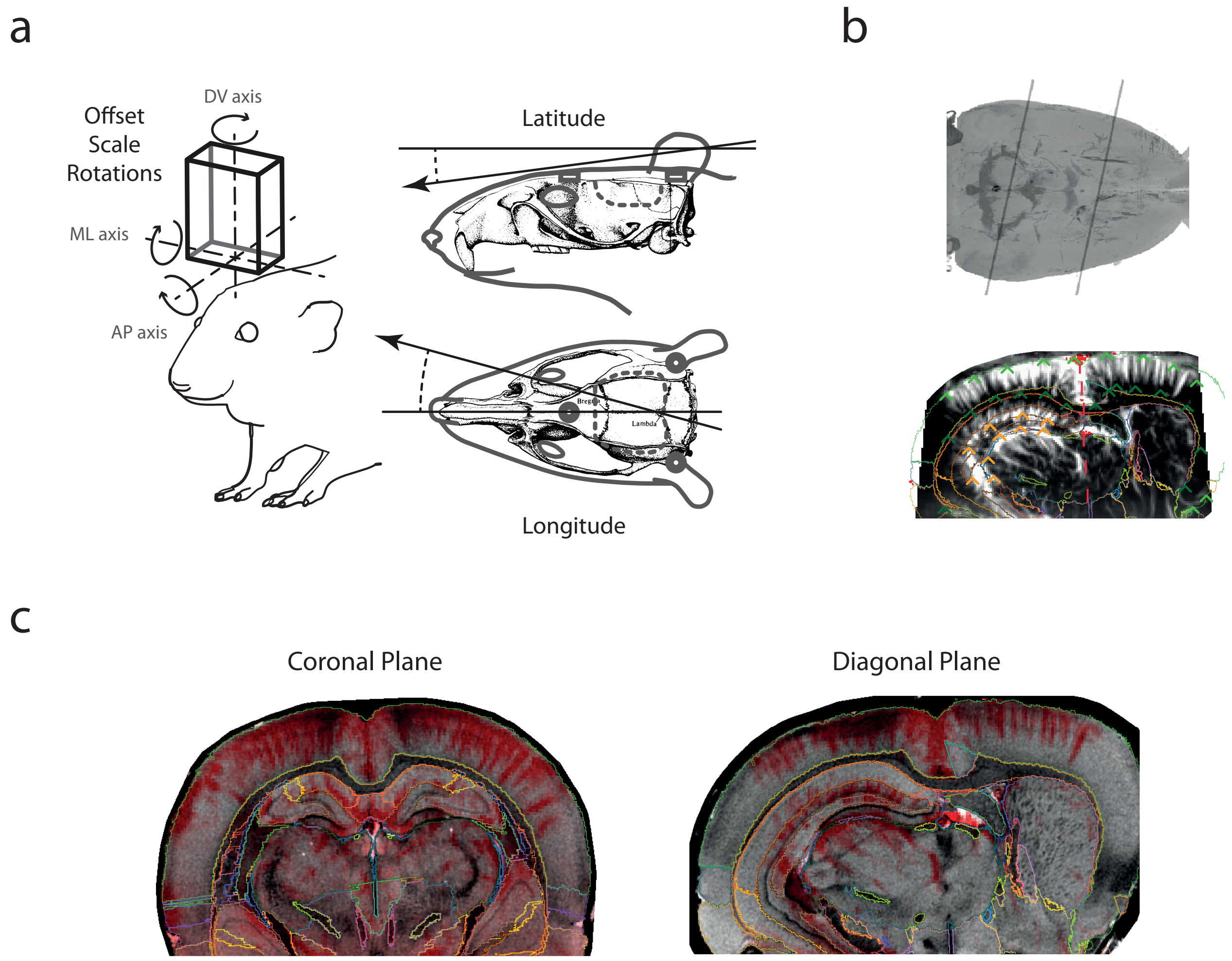

Supplementary Figure 3: Waxholm-based atlas registration of vascular planes

(a) Atlas registration of vascular images is performed by finding the physical parameters of probe and probe holder orientation to acquire vascular planes. We listed 3 parameters for each axis (1 rotation, 1 offset value and 1 scaling value). In multi-plane recordings, we added 2 angles (latitude and longitude) corresponding to the probe holder axis and 2 values (offset and scale) corresponding to consecutive-plane spacing. Adapted from reference [3], Copyright (1985), with permission from Elsevier.
(b) Top - Example of two diagonal planes superimposed on volumetric data. Bottom - We labeled vascular images with anatomically-salient landmarks including outer cortex edge (dark green), inner cortex edge (light green), dentate gyrus edge (orange) and brain mid-plane axis (red). These landmarks are used to minimize a global mean-square error between landmarks and corresponding region edge.
(c) Our algorithm allows for registration of any vascular plane onto Waxholm atlas[4]. We give two resulting registrations for a coronal plane (left) and a diagonal plane including whole hippocampus (right).

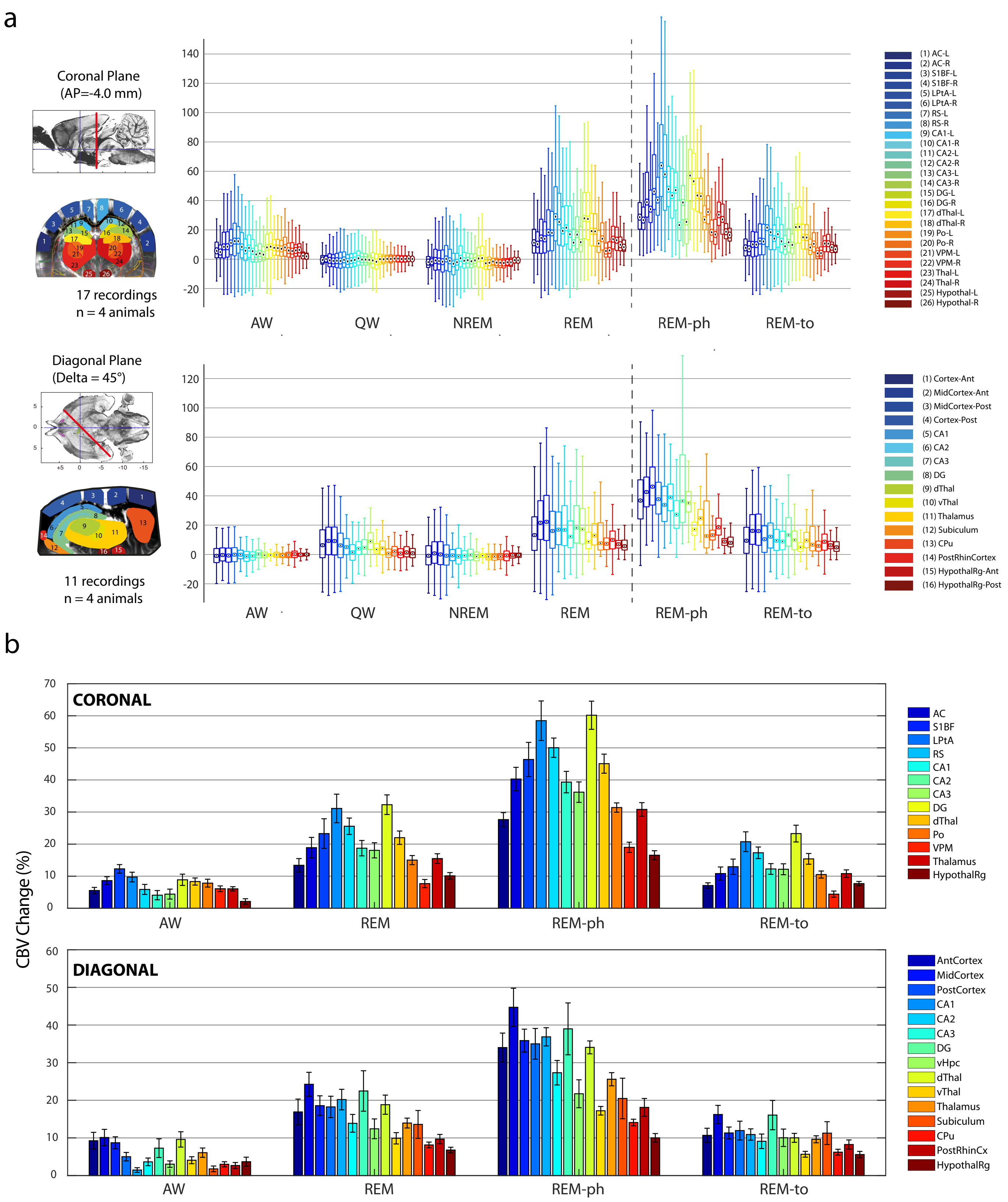

Supplementary Figure 4: Details of vascular amplification during REMS

(a) Whisker plot distributions of regional CBV averages for one coronal plane (AP = -4.0 mm, 17 recordings, 4 animals) and one diagonal plane (45° rotation along dorso-ventral axis, 11 recordings, 4 animals), for all 4 periods described previously. REM sleep is divided in phasic (REM-ph) and tonic (REM-to) vascular. Note that both REM and REM-ph distributions are consistently separated from AW distribution. Left panels adapted from reference [4], Copyright (2014), with permission from Elsevier.

(b) Details of mean vascular amplification (Figure 2C) for all regions and recordings along the typical coronal (Top) and diagonal planes (Bottom). Error bars show standard-error of the mean.

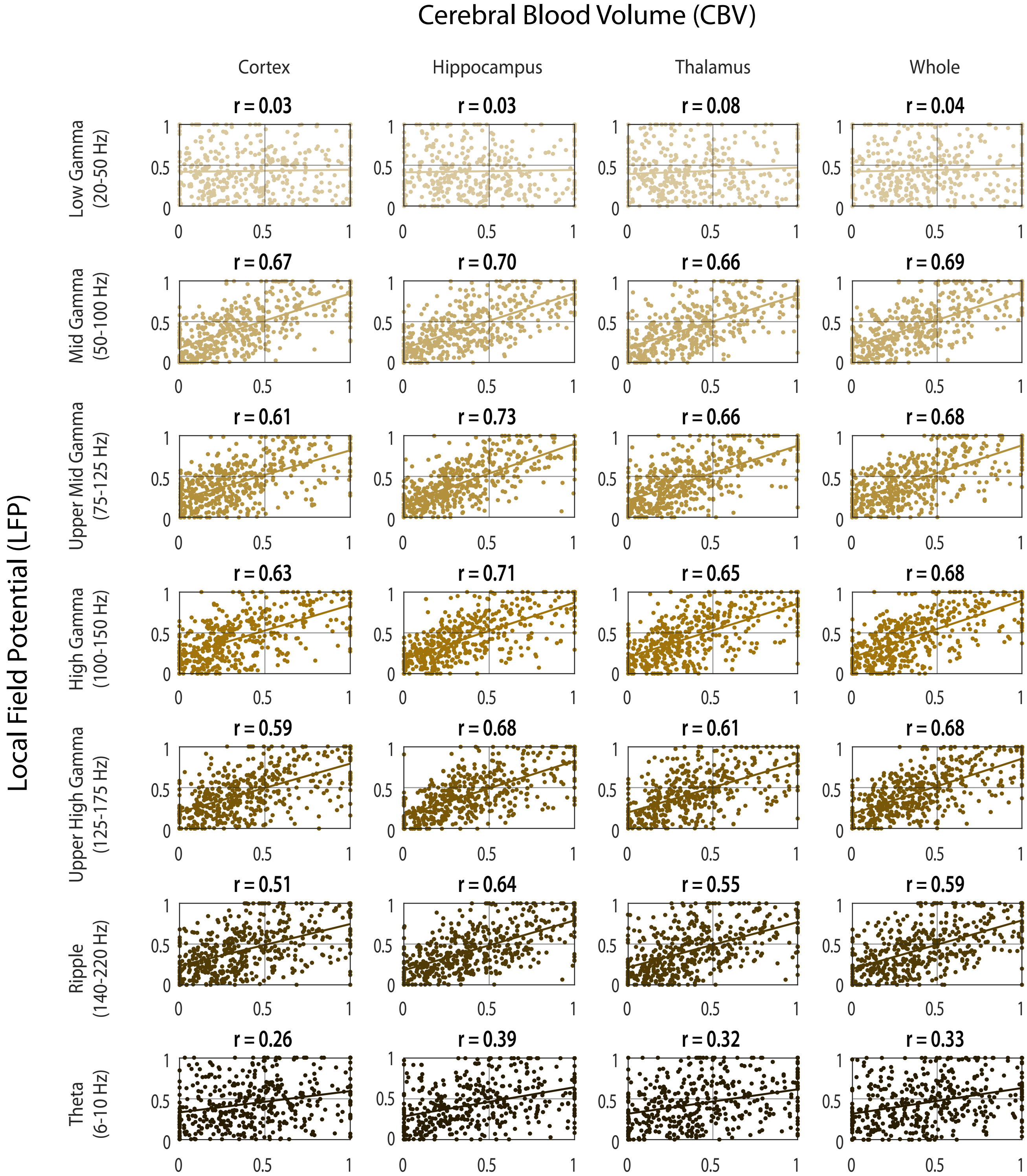

Supplementary Figure 5: Details of CBV-LFP correlations

Details of the full LFP-CBV raster plot for all recordings as shown in Figure 3C.
We displayed four main regions including cortex, dorsal hippocampus, thalamus and whole brain. This shows that low-gamma and ripple bands poorly correlate with the vascular events observed during REM sleep, whereas theta, mid and high gamma accurately strongly correlate with vascular surge amplitude. Note that the strongest correlations are obtained for the second (or third row) and second column, that is when comparing LFP fast gamma oscillations and hemodynamics within the hippocampus.

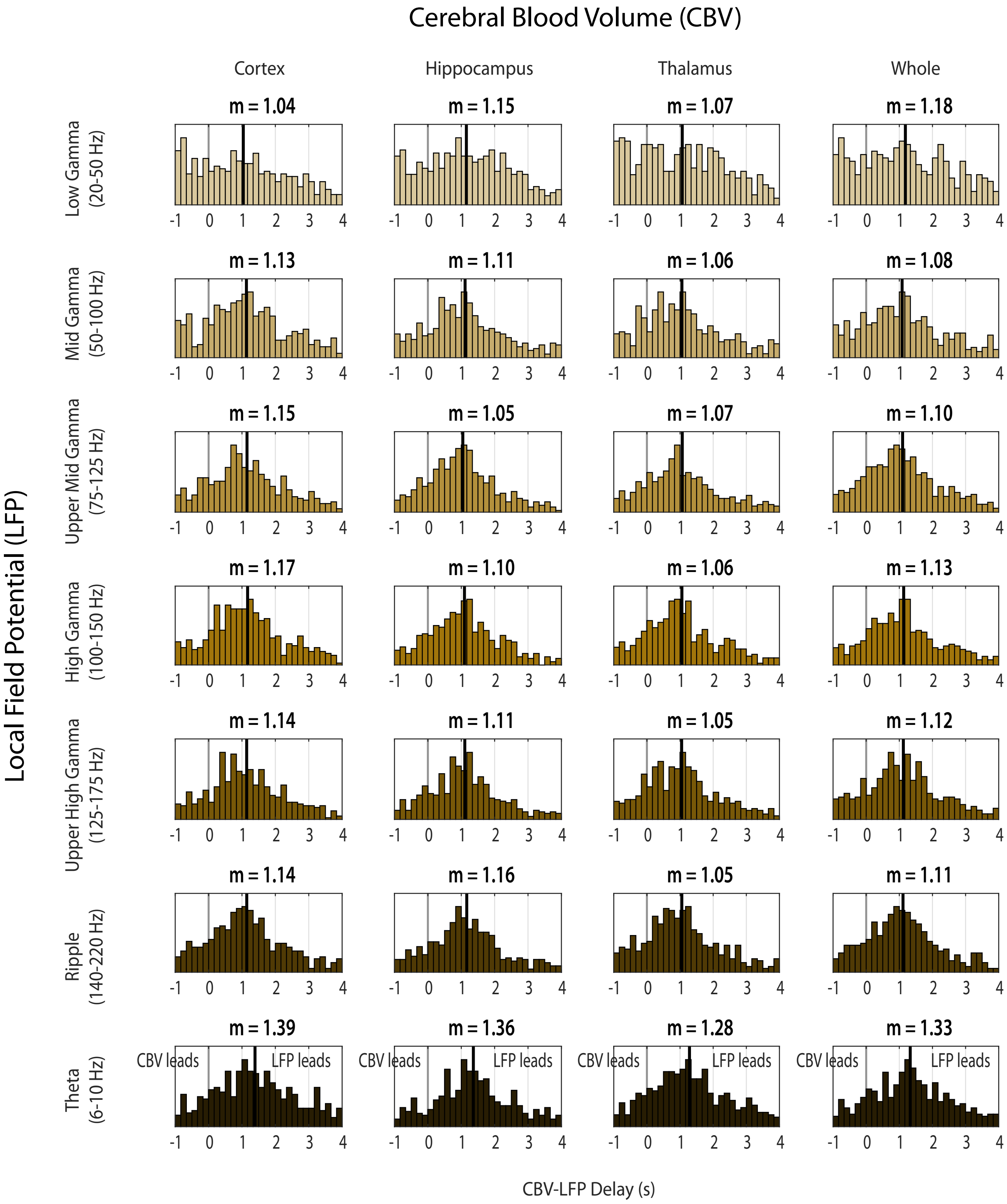

Supplementary Figure 6: Details of CBV-LFP delays

Details of the full LFP-CBV delay histograms for all recordings as shown in Figure 3E.
We displayed them for four main regions including cortex, dorsal hippocampus, thalamus and whole brain. This shows that the vast majority of electrophysiological events occur before vascular surges (all timing distributions are skewed towards positive delays, showing that LFP peaks lead CBV peaks). Apart from the low gamma band, all of these distributions show asymetry and a clear maximum, the strongest assymetry being observed for upper mid and high gamma bands. Note that delays differ across regions for all frequency bands, showing that vascular activity peaks earlier in the thalamus, then in the hippocampus and finally in the cortex.

HPC - CORTEX	QW		NREM			AW			REM		
Animal	IC_95%		IC_95%		Z_score	IC_95%		Z_score	IC_95%		Z_score
Rat1 (3 recordings)	0,562	0,676	0,603	0,658	0,29	0,828	0,883	10,60	0,910	0,945	13,57
Rat2 (4 recordings)	0,716	0,773	0,608	0,644	-10,05	0,782	0,830	-0,23	0,787	0,850	2,77
Rat3 (6 recordings)	0,596	0,722	0,643	0,698	0,24	0,680	0,794	1,87	0,763	0,836	4,63
Rat4 (3 recordings)	0,689	0,754	0,619	0,649	-5,00	0,673	0,737	-0,83	0,832	0,889	6,36
Average	0,641	0,731	0,618	0,662	-3,629	0,741	0,811	2,852	0,823	0,880	6,829

THAL-CORTEX	QW		NREM			AW			REM		
Animal	IC_95%		IC_95%		Z_score	IC_95%		Z_score	IC_95%		Z_score
Rat1 (3 recordings)	0,566	0,671	0,447	0,516	-4,58	0,863	0,909	13,10	0,884	0,927	11,53
Rat2 (4 recordings)	0,745	0,796	0,519	0,562	-14,83	0,732	0,783	-0,36	0,850	0,895	4,16
Rat3 (6 recordings)	0,443	0,600	0,502	0,573	0,36	0,643	0,774	3,79	0,755	0,831	7,64
Rat4 (3 recordings)	0,774	0,825	0,504	0,540	-15,61	0,825	0,863	3,27	0,846	0,899	4,13
Average	0,632	0,723	0,493	0,548	-8,665	0,766	0,832	4,950	0,834	0,888	6,865

HPC-THAL	QW		NREM			AW			REM		
Animal	IC_95%		IC_95%		Z_score	IC_95%		Z_score	IC_95%		Z_score
Rat1 (3 recordings)	0,566	0,696	0,573	0,626	-1,30	0,889	0,920	14,39	0,925	0,955	14,75
Rat2 (4 recordings)	0,623	0,701	0,486	0,530	-8,25	0,749	0,796	6,24	0,834	0,883	9,09
Rat3 (6 recordings)	0,483	0,638	0,558	0,625	0,77	0,731	0,836	5,19	0,887	0,926	13,28
Rat4 (3 recordings)	0,618	0,697	0,498	0,534	-6,62	0,714	0,770	3,91	0,906	0,939	13,45
Average	0,573	0,683	0,529	0,579	-3,846	0,771	0,831	7,431	0,888	0,926	12,644

Supplementary Table 1: Inter-individual Analysis of CBV coupling strength (expressed as a correlation coefficient) across 3 main brain regions and arousal states.

CBV levels are expressed in % of change relative to the baseline (3 first minutes of quiet wake in each recording). Z-scores refer to the distance of the corresponding distribution relative to the quiet wake.

Neocortex	AW	REM	REM-PHASIC
Recording 1	3.008	5.848	30.480
Recording 2	6.747	12.357	24.431
Recording 3	1.986	11.855	19.502
Recording 4	8.299	16.665	30.089
Recording 5	4.560	9.267	27.867
Recording 6	3.064	8.671	16.644
Recording 7	6.645	40.472	55.500
Recording 8	5.929	6.126	36.904
Recording 9	4.908	12.154	24.975
Recording 10	9.536	4.881	27.027
Recording 11	10.300	29.002	51.724
Recording 12	5.541	29.442	43.963
Recording 13	10.192	24.062	29.846
Recording 14	0.581	4.834	12.703
Recording 15	23.219	14.614	29.310
Recording 16	13.910	11.204	27.794
Recording 17	15.294	20.424	38.611
Mean	7.866	15.405	31.022
Sem	1.366	2.460	2.768

dHippocampus	AW	REM	REM-PHASIC
Recording 1	9.626	20.219	61.639
Recording 2	12.271	42.276	79.990
Recording 3	11.674	34.675	52.483
Recording 4	4.449	22.730	42.149
Recording 5	4.059	13.557	37.748
Recording 6	2.997	18.694	33.775
Recording 7	3.785	45.041	55.815
Recording 8	-1.773	12.476	55.134
Recording 9	2.193	16.104	33.584
Recording 10	8.131	15.183	51.607
Recording 11	15.813	40.818	69.387
Recording 12	-0.833	30.338	44.647
Recording 13	-0.930	23.779	29.651
Recording 14	7.464	28.564	58.568
Recording 15	13.048	33.280	67.641
Recording 16	6.495	27.676	60.302
Recording 17	17.993	31.303	60.785
Mean	6.851	26.865	52.641
Sem	1.433	2.481	3.406

dThalamus	AW	REM	REM-PHASIC
Recording 1	8.762	18.316	54.941
Recording 2	12.917	32.557	68.798
Recording 3	8.731	30.543	43.434
Recording 4	7.155	31.203	51.625
Recording 5	6.231	19.837	45.270
Recording 6	11.328	23.178	38.810
Recording 7	-0.865	31.003	39.728
Recording 8	5.324	11.412	52.606
Recording 9	0.688	6.427	18.130
Recording 10	5.541	8.922	32.888
Recording 11	16.986	33.620	63.447
Recording 12	8.884	25.773	36.119
Recording 13	NaN	NaN	NaN
Recording 14	8.584	21.206	41.168
Recording 15	11.014	20.181	47.593
Recording 16	7.629	14.573	36.244
Recording 17	14.391	23.215	50.378
Mean	8.331	21.998	45.074
Sem	1.113	2.082	2.971

Whole	AW	REM	REM-PHASIC
Recording 1	6.115	12.676	38.490
Recording 2	9.142	23.423	48.499
Recording 3	7.266	25.292	36.109
Recording 4	7.079	21.505	34.782
Recording 5	5.924	14.021	35.191
Recording 6	7.921	16.650	27.663
Recording 7	1.470	22.758	28.214
Recording 8	6.403	8.443	36.215
Recording 9	0.571	5.848	14.317
Recording 10	5.075	7.187	27.455
Recording 11	10.678	22.455	40.694
Recording 12	6.087	20.971	29.032
Recording 13	4.263	13.029	15.818
Recording 14	2.579	9.738	22.278
Recording 15	6.656	13.296	30.826
Recording 16	5.901	10.121	24.893
Recording 17	9.927	15.877	33.400
Mean	6.062	15.488	30.816
Sem	0.664	1.513	2.104

Supplementary Table 2: Details of vascular amplification for all coronal recordings

For each recording, we computed the average CBV level in four regions including neocortex, dorsal hippocampus, dorsal thalamus and whole thalamus. The values displayed in Fig. 2C are extracted from these tables. CBV units are given in % of change relative to the baseline.

Neocortex	AW	REM	REM-PHASIC
Recording 1	1.513	13.099	21.718
Recording 2	3.559	8.776	23.555
Recording 3	5.781	2.401	16.370
Recording 4	2.184	25.775	35.493
Recording 5	8.953	25.886	41.055
Recording 6	3.325	13.853	23.738
Recording 7	9.766	9.453	20.340
Recording 8	11.738	20.162	35.718
Recording 9	10.963	17.345	23.229
Recording 10	0.960	11.675	20.243
Recording 11	13.587	15.746	43.191
Mean	6.575	14.925	27.696
Sem	1.374	2.156	2.813

Thalamus	AW	REM	REM-PHASIC
Recording 1	4.203	16.864	28.199
Recording 2	6.478	11.578	28.341
Recording 3	4.756	6.839	18.806
Recording 4	1.130	16.982	22.733
Recording 5	4.285	14.098	23.239
Recording 6	12.500	21.996	31.330
Recording 7	4.817	8.958	23.627
Recording 8	12.417	17.096	30.575
Recording 9	6.447	11.911	18.111
Recording 10	-0.050	11.279	20.230
Recording 11	9.907	15.870	36.385
Mean	6.081	13.952	25.598
Sem	1.238	1.309	1.750

dHippocampus	AW	REM	REM-PHASIC
Recording 1	0.520	23.790	39.739
Recording 2	2.820	13.210	41.711
Recording 3	4.125	4.231	26.929
Recording 4	0.298	21.945	31.976
Recording 5	5.848	23.223	38.773
Recording 6	11.878	37.260	56.358
Recording 7	8.347	12.161	33.920
Recording 8	6.680	20.367	38.534
Recording 9	10.780	29.013	43.598
Recording 10	2.150	27.330	43.404
Recording 11	15.452	31.793	70.070
Mean	6.264	22.211	42.274
Sem	1.486	2.865	3.591

vHippocampus	AW	REM	REM-PHASIC
Recording 1	0.214	0.341	1.505
Recording 2	1.038	3.316	9.173
Recording 3	1.143	4.272	15.275
Recording 4	3.471	11.793	15.163
Recording 5	NaN	NaN	NaN
Recording 6	NaN	NaN	NaN
Recording 7	3.952	9.985	23.396
Recording 8	7.564	16.770	27.066
Recording 9	3.672	23.218	31.314
Recording 10	-0.599	24.362	34.056
Recording 11	6.834	17.744	38.646
Mean	3.032	12.422	21.733
Sem	0.860	2.629	3.712

Supplementary Table 3: Details of vascular amplification for all diagonal recordings

For each recording, we computed the average CBV level in four regions including neocortex, ventral and dorsal hippocampus, and whole thalamus. The values displayed in Figure 2C are extracted from these tables. CBV units are expressed in % of change relative to the baseline.

		LFP			CBV			
Δt	# Surges	Gamma-low	Gamma-mid	Gamma-high	Thalamus	Hippocampus	Cortex	Whole
Recording 1	4	0.663	0.513	0.175	2.474	2.724	2.349	2.599
Recording 2	9	0.875	0.743	0.206	2.564	2.508	3.397	2.731
Recording 3	6	0.150	0.150	-0.120	2.537	2.453	2.536	2.536
Recording 4	5	NaN	1.267	0.317	3.085	2.885	3.285	3.085
Recording 5	2	NaN	-1.225	-0.925	1.642	1.892	1.892	1.892
Recording 6	1	NaN	NaN	0.000	2.202	2.202	3.202	1.702
Recording 7	2	NaN	-2.300	-1.100	1.428	1.178	0.428	0.428
Recording 8	5	0.250	0.063	-0.150	1.596	1.496	1.896	1.696
Recording 9	9	1.963	0.557	0.600	1.931	1.931	2.155	2.099
Recording 10	23	-0.265	0.388	0.321	1.014	1.306	1.270	1.270
Recording 11	13	-0.475	-0.258	0.268	0.970	1.067	1.003	0.906
Recording 12	16	0.956	0.107	0.180	1.525	1.749	1.693	1.833
Recording 13	27	0.543	0.490	0.475	1.382	1.581	1.647	1.514
Recording 14	12	0.538	0.585	0.111	1.554	1.514	1.594	1.554
Recording 15	18	1.370	0.900	0.320	1.302	1.595	1.408	1.541
Recording 16	29	0.190	0.079	0.570	1.015	1.280	1.015	1.065
Recording 17	17	-0.196	0.368	0.156	1.036	1.149	1.459	1.374
Recording 18	10	-0.658	-0.100	-0.294	0.682	1.402	0.730	0.922
Recording 19	8	1.317	0.767	0.392	1.590	1.710	2.009	1.830
Recording 20	11	-1.242	0.178	-0.006	0.803	0.934	0.847	0.891
Recording 21	13	0.550	1.136	0.367	1.338	1.485	1.596	1.485
Recording 22	41	0.421	-0.015	0.227	1.152	1.304	1.140	1.152
Recording 23	20	-0.208	0.426	0.308	0.850	0.946	1.354	1.114
Recording 24	50	0.394	0.584	0.432	1.268	1.364	1.518	1.422
Mean		0.3	0.34	0.281	1.303	1.458	1.493	1.438
St-dev		1.655	1.39	1.084	1.16	1.206	1.272	1.195
Z-score		3.402	4.579	4.854	21.056	22.653	21.985	22.539
Sem (ms)		7.5	4.9	3.6	3.3	3.4	3.6	3.6

Supplementary Table 4: Details of timings of LFP and CBV events relative to the peak of theta burst

For each recording, we isolated 351 surges displaying a prominent peak in all three regions (cortex, dorsal hippocampus and thalamus). We extracted relative timing compared to the peak of theta activity preceding each vascular surge.

Supplementary References

1. Kjonigsen, L. J., Lillehaug, S., Bjaalie, J. G., Witter, M. P. & Leergaard, T. B. Waxholm Space atlas of the rat brain hippocampal region: Three-dimensional delineations based on magnetic resonance and diffusion tensor imaging. *NeuroImage* **108**, 441–449 (2015).
2. Belluscio, M. A., Mizuseki, K., Schmidt, R., Kempter, R. & Buzsaki, G. Cross-Frequency Phase-Phase Coupling between Theta and Gamma Oscillations in the Hippocampus. *Journal of Neuroscience* **32**, 423–435 (2012).
3. Paxinos, G., Watson, C., Pennisi, M. & Topple, A. Bregma, lambda and the interaural midpoint in stereotaxic surgery with rats of different sex, strain and weight. *Journal of Neuroscience Methods* **13**, 139–143 (1985).
4. Papp, E. A., Leergaard, T. B., Calabrese, E., Johnson, G. A. & Bjaalie, J. G. Waxholm Space atlas of the Sprague Dawley rat brain. *NeuroImage* **97**, 374–386 (2014).
