# Supplementary material for: Local hippocampal fast gamma rhythms precede brain-wide hyperemic patterns during spontaneous rodent REM sleep
Source: Nat Commun. 2018 Dec 18;9:5364. doi: 10.1038/s41467-018-07752-3 (PMC6299136; doi:10.1038/s41467-018-07752-3)
Supplement: Supplementary file 6 — Description of Additional Supplementary Files [file 41467_2018_7752_MOESM6_ESM.pdf]

Description of Additional Supplementary Files

File Name: Supplementary Movie 1
Description: Typical transition between non-REM sleep (NREMS) and REM sleep (REMS) captured by functional Ultrasound (fUS) and EEG - Rat #1 - Coronal plane - Bregma = -4.0 mm

File Name: Supplementary Movie 2
Description: Typical transition between non-REM sleep (NREMS) and REM sleep (REMS) captured by functional Ultrasound (fUS) and EEG - Rat #2 - Coronal plane - Bregma = -6.0 mm

File Name: Supplementary Movie 3
Description: Typical transition between non-REM sleep (NREMS) and REM sleep (REMS) captured by functional Ultrasound (fUS) and EEG - Rat #3 - Diagonal plane - Delta = 45° from sagittal plane.
